# Combination of hearing screening and genetic screening for deafness-susceptibility genes in newborns

**DOI:** 10.3892/etm.2013.1406

**Published:** 2013-11-12

**Authors:** GEN-DONG YAO, SHOU-XIA LI, DING-LI CHEN, HAI-QIN FENG, SU-BIN ZHAO, YONG-JIE LIU, LI-LI GUO, ZHI-MING YANG, XIAO-FANG ZHANG, CAI-XIA SUN, ZE-HUI WANG, WEI-YONG ZHANG

**Affiliations:** Department of Clinical Laboratory, Handan Central Hospital, Handan, Hebei 056002, P.R. China

**Keywords:** newborn, hearing screening, gene screening, MassARRAY platform, mitochondrial 12S rRNA, GJB2 gene, SLC26A4 gene

## Abstract

The aim of this study was to determine the clinical significance of the results of screening of newborn hearing and the incidence of deafness-susceptibility genes. One thousand newborn babies in the Handan Center Hospital (Handan, China) underwent screening of hearing and deafness-susceptibility genes. The first screening test was carried out using otoacoustic emissions (OAEs). Babies with hearing loss who failed to pass the initial screening were scheduled for rescreening at 42 days after birth. Cord blood was used for the screening of deafness-susceptibility genes, namely the GJB2, SLC26A4 and mitochondrial 12S rRNA (MTRNR1) genes. Among the 1,000 neonates that underwent the first hearing screening, 25 exhibited left-sided hearing loss, 21 exhibited right-sided hearing loss and 15 cases had binaural hearing loss. After rescreening 42 days later, only one of the initial 61 cases exhibited hearing loss under OAE testing. The neonatal deafness gene tests showed two cases with 1555A>G mutation and two cases with 1494C>T mutation of the MTRNR1 gene. In the SLC26A4 gene screening, four cases exhibited the heterozygous IVS7-2A>G mutation and one case exhibited heterozygous 1226G>A mutation. In the GJB2 gene screening, two cases exhibited the homozygous 427C>T mutation and 10 exhibited the heterozygous 235delC mutation. The genetic screening revealed 21 newborns with mutations in the three deafness-susceptibility genes. The overall carrier rate was 2.1% (21/1,000). The association of hearing and gene screening may be the promising screening strategy for the diagnosis of hearing loss.

## Introduction

Hearing loss is the most common human birth defect, with an incidence of approximately one case among 1,000 newborns. The American newborn hearing screening project began in 1964 and has gradually spread globally. Newborn hearing screening has been gradually implemented in large and middle-sized cities across China and an increasing number of children with hearing loss are diagnosed shortly after birth. The program has a profound effect on the language development, communication, cognition, mental health and career planning of the children if early intervention is achieved (1).

With the launch of newborn hearing screening, assessment of its effectiveness reveals its limitations. First, the major screening target is permanent hearing loss of >35 dB, which is not detected if the newborns have low-grade hearing loss. Secondly, late-onset or progressive hearing loss is not detected since newborn hearing is normal at birth. Cytomegalovirus infection, Pendred syndrome, autosomal dominant nonsyndromic hearing loss, recessive vestibular aqueduct expansion and mitochondrial DNA (mtDNA) mutations (such as 12S rRNA gene 1555A>G and 1494C>T) also lead to undetected hearing loss at birth, whereas late-onset hearing loss occurs later (2).

The development of molecular genetics has demonstrated that 50% of nonsyndromic deafness has genetic factors, which makes genetic testing a powerful weapon for screening children with hearing loss (3). A preliminary survey of Chinese domestic genetic epidemiology for deafness showed that the GJB2, SLC26A4 and mitochondrial 12S rRNA (MTRNR1) genes are common mutation hot spots in Chinese nonsyndromic hearing loss (4). Association detection of these three genes indicated that 26.65% of the Northern Chinese population are prelingually deaf (5). Therefore, association detection of GJB2, SLC26A4 and mitochondrial 12S rRNA was combined with hearing screening to determine the common sites and frequencies of newborn deafness gene mutation, which may be used to develop a more effective and earlier intervention for hearing disorders.

## Materials and methods

### Subjects

One thousand newborns in the Handan Center Hospital (Handan, China) between November 2010 and October 2011 were studied. The GJB2, SLC26A4 and MTRNR1 genes were tested simultaneously in 532 males and 468 females. This study was conducted in accordance with the Declaration of Helsinki and with approval from the Research Ethics Committee of the Central Hospital of Handan City (Handan, China). Written informed consent was obtained from all guardians/parents of the participants.

### Hearing screening

Hearing screening was performed in the maternity ward and the newborns were tested under quiet natural sleeping conditions using ambient noise <30 dB. An AccuScreen Screening Instrument (Madsen, Copenhagen, Denmark) was used for the hearing test. At the third day after birth, every newborn was tested at different frequencies and volumes using otoacoustic emissions (OAEs). If the newborn failed the test, the OAE test was repeated at 42 days after birth in a hearing diagnostic laboratory.

### Genetic information and blood samples

Approximately 2 ml of cord blood was collected from each newborn and then stored in an EDTA-anticoagulated vacutainer for gene screening (6). The parents were asked to provide information, including names, age, hospital number, home address, telephone number, pregnancy information, family genetic history, neonatal gender, weight and birth information.

### Deafness gene screening

DNA was extracted from the cord blood to screen for the GJB2, SLC26A4 and MTRNR1 genes. A MassARRAY system (Sequenom Inc., San Diego, CA, USA) was used to screen for the deafness mutation sites of GJB2 and SLC26A4 genes. The MTRNR1 gene was screened for the 1449C>T and 1555A>G mutation sites through direct sequencing.

Genomic DNA was extracted using a kit (Axygen Biotechnology Co., Ltd., Silicon Valley, CA, USA) according to the manufacturer’s instructions. A NanoDrop 1000 spectrophotometer (Thermo Fisher Scientific Inc., Wilmington, DE, USA) was used to detect the concentration and purity of the extracted DNA. Following completion of the extraction process, a final concentration of 10 ng/μl genomic DNA was achieved from all samples in the 384-well plates and preserved at −20°C.

### Genetic typing of the GJB2 and SLC26A4 genes

The Assay Designer package (Sequenom Inc., San Diego, CA, USA) was used to design the polymerase chain reaction (PCR) primers and single-base extension primers for the deafness loci on the GJB2 and SLC26A4 genes. The primer probe design was strictly in accordance with the requirements of the MassARRAY system and the probes were synthesized by Shanghai Invitrogen Biotechnology Co., Ltd. (Shanghai, China). A 14-plex PCR amplification reaction was performed in one well, and the detected point mutations are shown in Table I.

Genomic DNA (1 μl; 10 ng/μl) and 4 μl of PCR mixture (Sequenom Inc.) were added into 384-well plates. The reaction conditions were as follows: 95°C for 2 min; 95°C for 30 sec, 56°C for 30 sec and 72°C for 1 min, for 45 cycles; then 72°C for 5 min.

Shrimp alkaline phosphatase (SAP; New England Biolabs Inc., Beverly, MA, USA) reaction mixture (2 μl) was added into 384-well plates for the SAP reaction. The reaction conditions were as follows: 37°C for 40 min and 85°C for 5 min. After completing the reaction, the mixture was cooled to room temperature and stored at 4°C.

The extension reaction conditions were as follows: 94°C for 30 sec; 94°C for 5 sec, 52°C for 5 sec, 80°C for 5 sec, for 40 cycles; 72°C for 3 min. The products were then preserved at 4°C.

Approximately 16 μl of deionized water and 6 mg of resin were added to the 384-well plates for desalination and then were analyzed with matrix-assisted laser desorption/ionization-time of flight mass spectrometry (MALDI-TOF-MS). Final results were read by the MassARRAY RT real-time sotware sustems. Genotype analyses were completed by the MassARRAY Typer software.

### Sanger sequencing of the MTRNR1 gene

The two mutation points of the MTRNR1 gene, 1494C>T and 1555A>G, were combined to design the primers. The online software Primer3 (Whitehead Institute for Biomedical Research, Cambridge, MA, USA) was used for designing the mitochondrial amplification primers (http://bioinfo.ut.ee/primer3-0.4.0/primer3/. Accessed: October 23, 2013). The following parameters were set to improve the effectiveness and specificity of PCR amplification: the GC content in the primers was set between 40 and 60%, the annealing temperature was set between 55 and 60°C, the 3′-end of the primers were generally set to end with G or C to improve the integration of the primers into the template strand, and the amplified fragment was generally set between 200 and 500 bp to facilitate sequencing. The sequences of the mitochondrial primers were as follows: forward primer, 5′-CAACCTCACCACCTCTTGCT-3′ and reverse, 5′-GTAAGGTGGAGTGGGTTTGG-3′. The fragment length was 497 bp. The primers were synthesized by Shanghai Invitrogen Biotechnology Co., Ltd.

**Figure 1:**
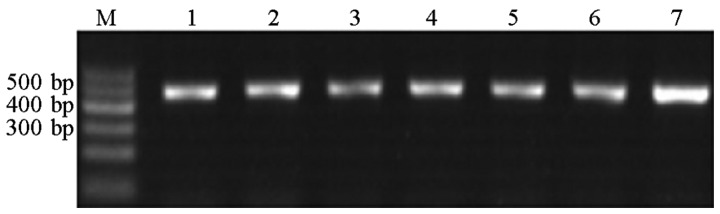
Results of PCR amplification: M: 600 marker; Lanes 1–7: the 12S rRNA gene fragment.

The PCR conditions were as follows: predenaturation at 94°C of 5 min; 35 cycles of 94°C for 30 sec, annealing at 60°C for 30 sec and extension at 72°C of 35 sec; and a final extension at 72°C for 5 min. Agarose gel electrophoresis (1.5% gel) was used to verify the results of the PCR amplification. The bands obtained were clearly visible and did not overlap (Fig. 1).

SAP mixture (1.5 μl) and the PCR product (1 μl) were added to 384-well plates. The reaction conditions were as follows: 37°C for 60 min and 80°C for 20 min. After the completion of the reaction, the reaction mixture was cooled to room temperature and stored at 4°C.

BigDye Terminator (BDT; 0.5 μl) and a DNA sequencing of the PCR-amplified product was performed bidirectionally on an ABI3730XL automated sequencer (Applied Biosystems, Foaster City, CA, USA) using the same primers. Sequence data were analyzed by evaluating samples for alignment with the National Center for Biotechnology Information reference (NCBI) sequence of MTRNR1 (NC_012920.1) using Sequencher Demo 3.0 and Mutation Surveyor Demo V4.0.

## Results

### Summary of results

Among the 1,000 newborns tested, 996 cases were rescreened. Only one of the 61 cases who failed the initial hearing screening test also failed the single-ear rescreening test. All 61 cases were screened for the three genes, and only the infant who failed the rescreening exhibited homozygous 427C>T mutation of the GJB2 gene. All other subjects passed the genetic screening, which indicated that no disease-causing mutation was present.

The two cases with the 1555A>G mutation and the two cases with the 1494C>T mutation of MTRNR1 and the other 16 cases that carried pathogenic mutations in the GJB2 and SLC26A4 genes passed the newborn hearing screening test.

### Hearing screening

In the initial screening of the hearing of 1,000 newborns, 939 (93.9%) of the newborns passed the initial OAE screening, whereas 25 cases failed the left-ear hearing test and 21 cases failed the right-ear hearing test, for total of 46 (4.6%) cases. Fifteen (1.5%) cases failed both the right- and the left-ear hearing tests. A total of 61 (6.1%) newborns failed the initial screening. At 42 days after birth, only one of the 61 cases who failed the initial hearing screening also failed the single-ear rescreening. Thus, one (0.1%) case did not pass OAE rescreening.

### Genetic screening

A total of 1,000 newborns were screened for mutations in the GJB2, SLC26A4 and MTRNR1 genes. Ten cases exhibited a heterozygous 235delC mutation of the GJB2 gene, and two cases exhibited a homozygous 427C>T mutation. Four cases exhibited heterozygous IVS7-2A>G mutation of the SLC26A4 gene and one case exhibited heterozygous 1226G>A mutation. Two cases exhibited homogeneous 1494C>T mutation of the MTRNR1 gene and two cases exhibited homogeneous 1555A>G mutation. The overall carrier rate was 2.1% (21/1,000). The specific carrier rates of the three genes among the 1,000 cases were then analyzed.

**Figure 2:**
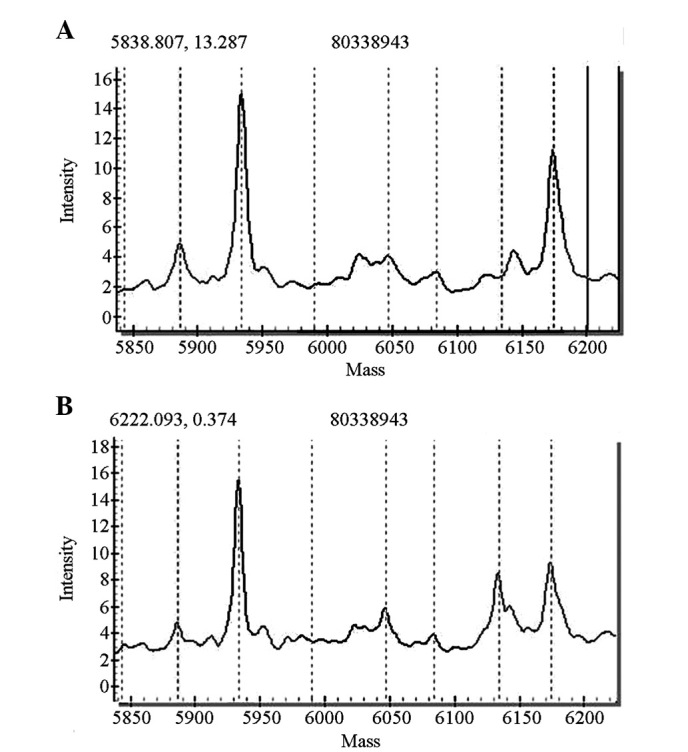
Results of MassARRAY detection of GJB2 235delC (A) wild-type and (B) mutant.

**Figure 3:**
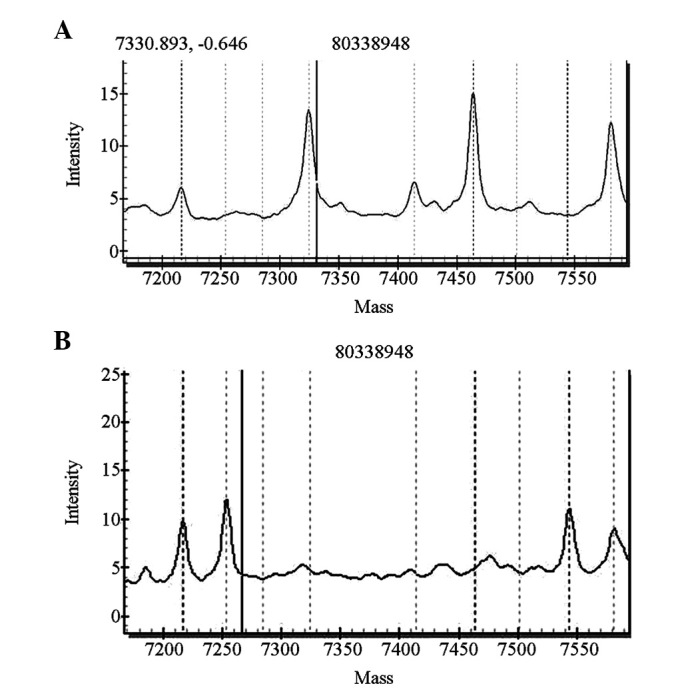
Results of MassARRAY detection of GJB2 427C>T (A) wild-type and (B) mutant.

GJB2 gene screening indicated that 10 cases had miscellaneous 235delC mutations and two cases had homozygous 427C>T mutations. The pathogenic carrier rate was 1.2% (12/1,000), among whom 11 cases passed the initial hearing screening test. One case did not pass the primary screening and the rescreening, and was identified to carry a homozygous 427C>T mutation (Figs. 2 and 3).

**Figure 4:**
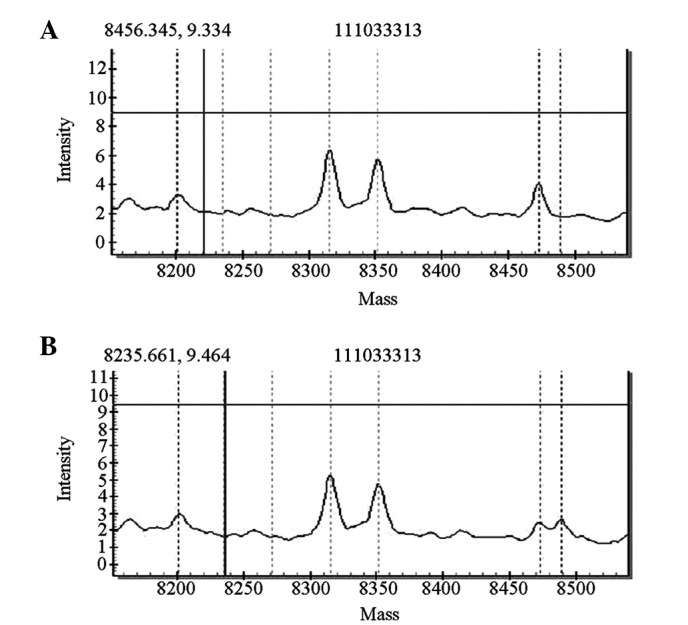
Results of MassARRAY detection of SLC26A4 IVS7-2A>G (A) wild-type and (B) mutant.

**Figure 5:**
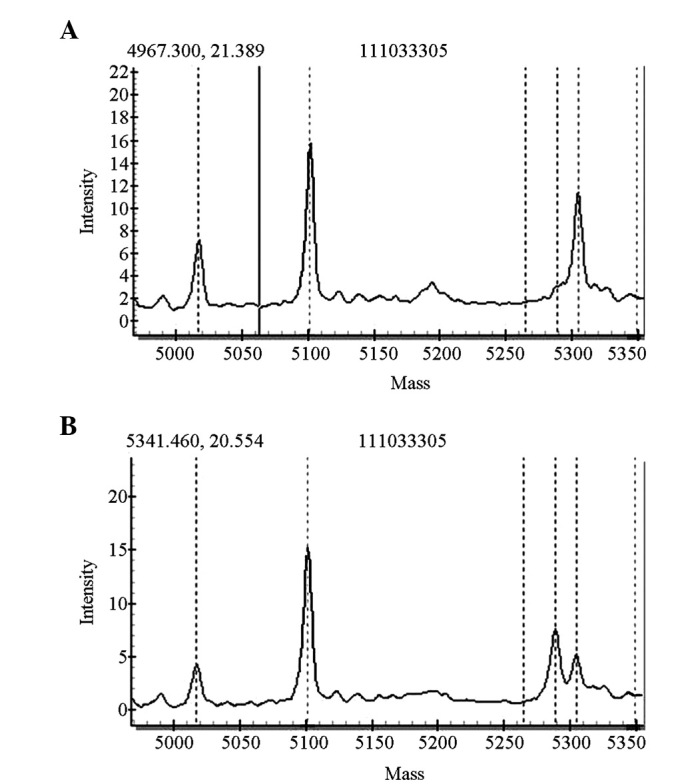
Results of MassARRAY detection of SLC26A4 1226G>A (A) wild- type and (B) mutant.

For the SLC26A4 gene screening, four cases carried heterozygous IVS7-2A>G mutations of the SLC26A4 gene and one case carried a heterozygous 1226G>A mutation. The pathogenic carrier rate was 0.5% (5/1,000). The five newborns with these mutations passed the initial hearing screening test. The distributions of the mutations are shown in Figs. 4 and 5.

**Figure 6:**
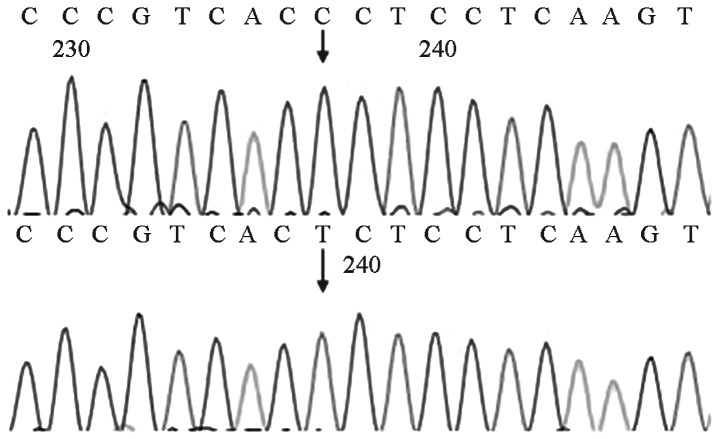
Identification of 1494C>T mutation of MTRNR1.

**Figure 7:**
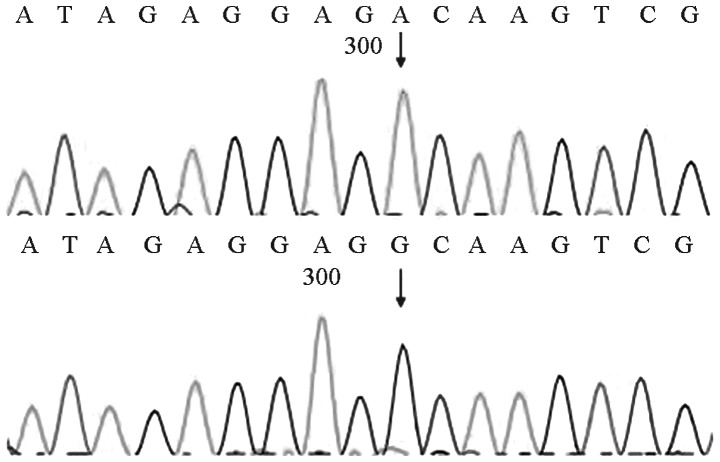
Identification of 1555A>G mutation of MTRNR1.

MTRNR1 genetic screening revealed that four of the 1,000 cases carried gene mutations [pathogenic carrier rate of 0.4% (4/1,000)]. Two of the four cases exhibited homozygous 1494C>T mutations, whereas the other two cases exhibited homozygous 1555A>G mutations. The mutations detected by the forward sequencing were identified via reverse sequencing. The four cases passed the initial hearing screening test. The forward sequencing results are shown in Figs. 6 and 7.

## Discussion

In the present study, a program for the simultaneous screening of newborn hearing and genes was implemented, that is, the hearing of the newborns was tested and GJB2, SLC26A4 and MTRNR1 genes were screened simultaneously. Of the 999 newborns that passed the newborn hearing screening, 20 exhibited mutations in deafness-associated genes. The carrier rate of disease-causing mutations reached 2.1%, which is 20 times higher than the incidence of congenital deafness (0.1%) from hearing screening. Notably, these cases were not identified through hearing screening. Ten cases exhibited a heterozygous mutation of GJB2 235delC (1%; 10/1,000) and all passed the hearing screening test. The possibility of late-onset type hearing loss among these 10 cases is significantly increased during development compared with that of newborns with the normal gene. Four cases of IVS7-2A>G heterozygous mutation were identified during SLC26A4 gene screening. Individuals with this mutation should avoid strenuous exercise, trauma and collision, and follow-up examinations should be conducted at regular intervals. The aforementioned 14 newborns should also avoid marriage with carriers of the same genotype since their offspring would have a 25% chance of deafness. Two cases exhibited homozygous 427C>T mutation of GJB2 and one of these cases failed the hearing screening test in 1998. The 427C>T mutation was first reported in the GJB2 gene, with the mutation causing the amino acid at position 143 to change from arginine to tryptophan, thereby causing autosomal recessive deafness (7). One case of heterozygous 1226G>A mutation of the SLC26A4 gene was observed; this mutation was first reported in 1998 (8). The arginine at position 409 is changed into histidine in the vestibular aqueduct expansion deafness phenotype, which is commonly associated with autosomal recessive deafness (8). Temporal bone computed tomography (CT) is recommended for individuals that carry the heterozygous mutation in the SLC26A4 gene to detect whether vestibular aqueduct enlargement has occurred, and regular follow-up examinations should be performed. In the present study, four cases of MTRNR1 gene mutations were also identified. Among them two cases had a 1555A>G mutation and two cases had a 1494C>T mutation. The MTRNR1 pathogenic mutation carrier rate was 0.4% (4/1,000). These four babies are extremely sensitive to aminoglycosides (including streptomycin, neomycin, kanamycin and gentamicin) and any exposure to these drugs is likely to lead to irreversible deafness. If the babies are not exposed to these drugs, head trauma, exposure to noisy environments and various infections that may cause hearing loss, they may have normal hearing during their lifetime. Due to the maternal transmission of mtDNA, these genetic test results may be treated as an early warning for maternal family members of these four cases. The carrier rate of pathogenic mutations in mitochondrial genes in this region is much higher than in other regions (9), which indicates the requirement for gene screening among the newborns from this region.

In the current newborn deafness gene screening program, a high-flux method was used for detecting gene mutations, namely, the MassARRAY system. The basic principle of the system is based on MALDI-TOF-MS technology, combined with a highly specific and sensitive chip technology. The system facilitates the research and application of single-nucleotide polymorphism (SNP) genotyping, gene expression, copy number variation, gene methylation analysis, pathogen typing and prenatal diagnosis in one platform. The system integrates the high sensitivity of PCR and high accuracy of mass spectrometry (10). The advantageous feature of the system is its ability to perform rapid genotype identification with high accuracy and directly measure target DNA with SNPs or mutations (11). The MassARRAY system is non-hybrid dependent, free from potential hybrid mismatch interference, does not require various biomarkers, and completes a large number of loci detection and fully automatic analysis within a short time through its high-density Spectro CHIP lattice chip analysis system. We designed 14 reactions/well by selecting the mutation hot spots of the Chinese deafness genes. The selection of the MassARRAY system greatly reduced the screening cost, established a high-flux genetic mutation detection method, and provided a new application for clinical MassARRAY detection of deafness-associated mutations.

The present study initially explored the distribution of deafness-susceptibility genes in newborn hearing screening and analyzed the conditions of newborn deafness gene carriers. The results demonstrate the necessity and feasibility of genetic screening for deafness in newborns. Newborn hearing screening combined with deafness-susceptibility gene screening may be a promising strategy for the early diagnosis of prelingual hearing loss, for individuals with a high risk of delayed-type deafness or deafness gene carriers (7). In the present study, the total gene mutation carrier rate and the individual carrier rate of the three genes were extremely high. Therefore, deafness-susceptibility gene screening is important for identifying hereditary hearing loss. However, the genetic screening results do not provide accurate information regarding the hearing situation and prognosis of the newborns (12) due to numerous uncertainties and factors in the molecular detection results, including gene polymorphisms and single-locus heterozygous mutations. Therefore, hearing screening and genetic screening should be considered together.

## Figures and Tables

**Table I:** Fourteen SNPs used for genotyping.

Gene name	Mutation site	rs
GJB2	c.101T>C	rs35887622
	c.235delC	rs80338943
	c.592G>A	
	c.427C>T	rs80338948
SLC26A4	IVS7-2A>G	rs111033313
	c.916-c.917insG	
	c.754T>C	
	c.281C>T	
	IVS15+5G>A	
	c.2027T>A	rs111033318
	c.2168A>G	rs121908362
	c.439A>G	
	c.1226G>A	rs111033305
	c.589G>A	rs111033380

rs, reference SNP ID number.
